# Thymidylate synthase predictive power is overcome by irinotecan combination therapy with S-1 for gastric cancer

**DOI:** 10.1038/sj.bjc.6602139

**Published:** 2004-08-31

**Authors:** W Ichikawa, T Takahashi, K Suto, T Yamashita, Z Nihei, Y Shirota, M Shimizu, Y Sasaki, R Hirayama

**Affiliations:** 1 Second Department of Surgery, Saitama Medical School, 38, Moro-Hongo, Moroyama-cho, Iruma-gun, Saitama 350-0495, Japan; 2 Department of Surgery, Tojun Hospital, 4-3-4, Hitotsuya, Adachi, Tokyo 121-0075, Japan; 3 Department of Pathology, Saitama Medical School, 38, Moro-Hongo, Moroyama-cho, Iruma-gun, Saitama 350-0495, Japan; 4 Department of Medical Oncology, Saitama Medical School, 38, Moro-Hongo, Moroyama-cho, Iruma-gun, Saitama 350-0495, Japan

**Keywords:** thymidylate synthase, dihydropyrimidine dehydrogenase, S-1, irinotecan, gastric cancer

## Abstract

The predictive values of thymidylate synthase (TS) and dihydropyrimidine dehydrogenase (DPD) gene expressions were retrospectively evaluated in patients with gastric cancer treated by a regimen containing S-1. The study population consisted of 53 patients registered into different two phase II studies for metastatic gastric cancer; 27 patients treated by S-1-alone study: 26 patients treated with S-1 combined with irinotecan (CPT-11). TS and DPD gene expressions in primary tumours were measured by the real-time reverse transcription PCR method. There was no statistical difference in DPD gene expression in terms of response in cases treated with S-1 alone and those treated with S-1 plus CPT-11. TS mRNA of responding tumours was lower than that of nonresponding ones when treated with S-1 (*P*<0.005). In the S-1-alone group, taking TS cutoff as the median values, the response rate in the low TS group was 50%, but only 8% in the high TS group (*P*<0.05). Patients with low TS gene expression survived longer than those with high TS gene expression (*P*<0.0001). However, there was no statistically significant difference in response rate and survival between patients with low TS tumours and those with high TS tumours, when the cutoff was taken as the median value of TS gene expression in the group treated with S-1 plus CPT-11. In conclusion, treatment effects of S-1 monotherapy for gastric cancer were determined by the status of TS gene expression, regardless of DPD gene expression. TS predictive power was overcome by CPT-11 combination therapy with S-1.

The main mode of action of 5-fluorouracil (5-FU) is thought to be through its active metabolite, 5-fluoro-deoxyuridine-monophosphate (FdUMP), which suppresses thymidylate synthase (TS), an essential DNA synthetic enzyme that catalyses the methylation of deoxyuridine monophosphate (dUMP) to deoxythymidine monophosphate (dTMP) (Langenbach *et al*, 1972; Peters *et al*, 1995). 5-FU is catabolised to 2-fluoro-*β*-alanine by dihydropyrimidine dehydrogenase (DPD), the first and rate-limiting enzyme (Heggie *et al*, 1987).

S-1 is a new oral fluorinated pyrimidine, in which tegafur (FT) has been combined with two 5-FU modulating substances: 5-chloro-2,4-dihydroxypyrimidine (gimeracil, CDHP), and potassium oxonate (oteracil potassium, Oxo), in a molar ratio of FT: CDHP: Oxo=1 : 0.4 : 1 (Shirasaka *et al*, 1996). FT is a prodrug of 5-fluorouracil (FU), which is absorbed after oral ingestion followed by conversion to 5-FU. CDHP reversibly inhibits the activity of DPD (Yamada *et al*, 2003), resulting in the increase of antitumour activity (Tatsumi *et al*, 1987). Two phase II studies of S-1 showed activity in gastric cancer, accompanied by mild-to-moderate toxicity. The response rate was 44–49% and the median survival time was 207–250 days, with 1- and 2-year survival rates of 36–37 and 14%, respectively (Sakata *et al*, 1998; Koizumi *et al*, 2000).

Irinotecan hydrochloride (CPT-11) is a water-soluble, semisynthetic derivative of camptothecin (CPT) that retains the original antitumour effects of CPT-11 due to the inhibition of DNA topoisomerase I (Topo-I) (Hsiang *et al*, 1989). CPT-11 was shown to lack cross-resistance with fluoropyrimidines in both experimental models and the clinical setting (Vanhoefer *et al*, 2001). The response rate of CPT-11 alone in gastric cancer was 23% in a Japanese phase II study (Futatsuki *et al*, 1994). The response rate in patients with prior 5-FU-containing regimens was 18.9%, which indicated a lack of cross-resistance between CPT-11 and 5-FU in gastric cancer. In our previous phase I study of S-1 and CPT-11 for metastatic gastric cancer, the recommended dose was 80 mg m^−2^ of CPT-11 on days 1 and 8 and 80 mg m^−2^ day^−1^ (40 mg m^–2^ b.i.d.) of S-1 for the first 2 weeks, repeated every 4 weeks (Yamashita *et al*, 2003).

In colorectal cancer, both intratumoural TS and DPD gene expression have been indicated to be positive predictive markers for the effectiveness of 5-FU or UFT combined with leucovorin (Salonga *et al*, 2000; Ichikawa *et al*, 2003). However, there is little information about the predictive values of TS or DPD gene expression in treatment with S-1-containing regimens. In this pilot study, we evaluated the predictive values of TS and DPD gene expressions in cases of metastatic gastric cancer treated with S-1 or S-1 combined with CPT-11, using formalin-fixed, paraffin-embedded tumour specimens.

## PATIENTS AND METHODS

### Clinical methods

The study population consisted of 53 patients with metastatic gastric cancer who received chemotherapy for metastatic disease after resection of primary tumours at the Second Department of Surgery, Saitama Medical School. All 53 patients were registered for two independent phase II studies; 27 patients for the phase II study of S-1 alone from January 1999 to March 2000, and 26 patients for the phase II study of S-1 combined with CPT-11 from January 2001 to December 2002.

In both phase II studies, no patients had received 5-FU chemotherapy preoperatively. Eligible patients for two phase II studies had (a) histologically proven gastric cancer with at least one measurable metastatic lesion; (b) Eastern Clinical Oncology Group scale performance status of 2 or better (Zubrod *et al*, 1960); (c) age of 80 years or younger; (d) no prior chemotherapy regimens for metastatic disease before entry; (e) adequate haematological, hepatic, and renal function; and (f) life expectancy of over 3 months. In patients treated with adjuvant chemotherapy, there was a washout period of at least 4 weeks. Patient characteristics are listed in Table 1

**Table 1:** Patient characteristics

	**S-1 alone**	**S-1 plus CPT-11**
No. of patients	27	26
		
*Age (years)*		
Median	58	61
Range	29–78	34–80
		
*Gender*		
Male/female	20/7	19/7
		
*PS*		
0/1/2	19/5/3	20/5/1
		
*Histology*		
Intestinal/diffuse	10/17	11/15
		
*Metastasis*		
Synchronous/metachronous	10/17	12/14
		
*Adjuvant chemotherapy*		
Yes/no	14/13	12/14
		
*Metastatic site*		
Liver	8	8
Lymph node	15	17
Lung	5	3
Others	5	8
		
*Second-line chemotherapy*		
Taxanes	5	10
Cisplatin+CPT-11	2	0
CPT-11	3	0

.

Before the treatment and after every two cycles of treatment, measurable disease was reassessed by computed tomography. Response evaluation was based on the standard UICC guidelines as complete response (CR), partial response (PR), no change (NC), or progressive disease (PD) (Hayward *et al*, 1978). There were one CR, seven PR, 13 NC, and six PD with a 29.6% (eight out of 27) response rate in the S-1-alone regimen (95% confidence interval, 13.8–50.2%), and two CR, 11 PR, nine NC, and four PD with a 50.0% (13 out of 26) response rate in the S-1 combined with CPT-11 regimen (95% confidence interval, 29.9–70.1%). All patients eventually died from cancer. The median survival times were 6.3 months, ranging from 2.1 to 23.0 months and 6.3 months ranging from 2.8 to 26.6 months for patients treated with S-1 and S-1 combined with CPT-11, respectively.

The S-1-alone regimen consisted of oral administration of S-1 (Taiho Pharmaceutical Co., Ltd., Tokyo, Japan) at 80 mg m^−2^ (standard dose) daily (40 mg m^−2^ b.i.d.) after meals. Three doses of S-1 were established according to body surface area (BSA) as follows: BSA <1.25 m^2^; 80 mg day^−1^; 1.25 m^2^⩽BSA <1.5 m^2^, 100 mg day^−1^; and 1.5m^2^ ⩽BSA, 120 mg day^−1^, as described previously (Sakata *et al*, 1998; Koizumi *et al*, 2000). One course consisted of consecutive administration for 28 days followed by 14 days of no drug administration.

The S-1 combined with CPT-11 comprised oral administration of S-1 at 80 mg m^−2^ day^−1^ (40 mg m^−2^ b.i.d.) during 2 weeks, with a 90-min infusion of 80 mg m^−2^ day^−1^ CPT-11 at days 1 and 8. Cycles were repeated every 4 weeks. Dose calculation of S-1 was performed in the same way as in the S-1-alone regimen. This regimen was decided according to previously reported phase I study (Yamashita *et al*, 2003).

These studies were approved by the Institutional Review Board of Saitama Medical School, and all patients gave written informed consent.

### Laboratory methods

#### Microdissection in primary tumours

A representative formalin-fixed, paraffin-embedded (FFPE) tumour specimen obtained from primary tumours was selected by a pathologist (MS) after examination of the haematoxylin- and eosin-stained slides. Sections 10 *μ*m in thickness were stained with nuclear Fast Red to enable visualisation of histology for laser capture microdissection (PALM Microlaser Technologies AG, Munich, Germany), which was performed to ensure that only tumour cells were studied. Microdissected samples were collected into a microcentrifuge tube.

#### RNA extraction and cDNA synthesis

RNA extraction and cDNA synthesis were done in Response Genetics Inc. (Los Angeles, CA, USA). RNA extraction was performed according to a proprietary procedure (US patent number 6,248,535) (Lord *et al*, 2000; Kornmann *et al*, 2003). Briefly, 600 *μ*l of xylene was added to each tube. After centrifugation for 7 min at 14 000 r.p.m., the supernatant was discarded, and the washing step was repeated three times. The deparaffinised materials were rehydrated in xylene : ethanol : water at the following ratios (95 : 95 : 5, 95 : 90 : 10, 95 : 80 : 20; 95 : 75 : 25, and 95 : 70 : 30). After each step, the rehydration medium was removed after centrifugation for 7 min at 14 000 r.p.m. After discarding the last supernatant, the pelleted sections were resolved in 70% ethanol. Then 400 *μ*l of buffer (4 M guanidine isothiocyanate solution including 0.5% sarcosine and 8 *μ*l 1 M DTT) were added to the dried tissue and homogenised mechanically. For RNA demodification, homogenates were heated at 95°C for 30 min. RNA was extracted from homogenates by addition of 50 *μ*l of 2 M sodium acetate (pH 4.0), 500 *μ*l of water-saturated phenol, and 100 *μ*l of chloroform–isoamyl mixture (49 : 1). RNA was recovered from the water phase by isopropanol precipitation and transferred to a new tube and precipitated with 10 *μ*l glycogen and 400 *μ*l isopropanol for 30 min at −20°C. After centrifuging for 7 min at 14 000 r.p.m., the pellet was washed with 500 *μ*l 75% ethanol. After drying, the pellet was dissolved in 50 *μ*l 5 mM Tris-HCl (pH 8.0). Reverse transcription was carried at 39°C for 45 min using 400 U of MMLV reverse transcriptase, 1 × first strand buffer, 0.04 *μ*g *μ*l^−1^ random hexamers, 10 mM DTT, and 1 mM deoxynucleoside triphosphate.

#### PCR quantification of mRNA expression

Target cDNA sequences were amplified by quantitative PCR using a fluorescence-based real-time detection method (ABI PRISM 7900 Sequence Detection System (Taqman); Applied Biosystems, Foster City, CA, USA) as previously described (Kornmann *et al*, 2003). Polymerase chain reaction was carried out for each gene of interest, and *β*-actin was used as an internal reference gene. The 25 *μ*l PCR reaction mixture contained 600 nmol l^−1^ of each primer, 200 nmol l^−1^ each of dATP, dCTP, and dGTP, 400 *μ*mol l^−1^ dUTP, 5.5 mmol l^−1^ MgCl_2_, and 1 × TaqMan buffer A containing a reference dye (all reagents were supplied by Applied Biosystems). The primers and probe sequences used were as follows: TS primers: GCCTCGGTGTGCCTTTCA and CCCGTGATGTGCGCAAT, probe 6FAM (carboxyfluorescein)-5′-TCGCCAGCTACGCCCTGCTCA-3′TAMRA (*N*,*N*,*N*′,*N*′-tetramethyl-6carboxyrhodamine); DPD primers: AGGACGCAAGGAGGGTTTG and GTCCGCCGAGTCCTTACTGA, probe 6FAM-5′- CAGTGCCTACAGTCTCGAGTCTGCCAGTG -3′TAMRA; *β*-actin primers: TGAGCGCGGCTACAGCTT and TCCTTAATGTCACGCACGATTT, probe 6FAM-5′- ACCACCACGGCCGAGCGG -3′TAMRA. The PCR conditions were 50°C for 10 s and 95°C for 10 min, followed by 42 cycles at 95°C for 15 s and 60°C for 1 min. Relative gene expression of TS and DPD was determined based on the threshold cycles of each gene in relation to the threshold cycle of the corresponding internal standard *β*-actin. The rise of the *β*-actin signal after cycle 37 using the described conditions indicated an insufficient amount of mRNA present for the subsequent TS and DPD quantitation.

The studies to validate these methods have been previously reported in colorectal cancer tissues (Kornmann *et al*, 2003). TS mRNA levels of paraffin-embedded material after complete pathological examination closely correlated with those of corresponding fresh-frozen tumour specimens obtained during surgery (*r*=0.70), although TS mRNA levels were ∼three-fold lower in paraffin-embedded tissues than in fresh-frozen tissues.

#### Statistics

Statistical analysis was performed using JMP software version 5.01 (SAS Institute, Inc., Cary, NC, USA). The Mann–Whitney *U* test was used to compare the responders and nonresponders in terms of the related gene expression. To evaluate the association with response, two-sided Fisher's exact test was used. Survival was calculated from the onset of chemotherapy until death. The overall survival curve was calculated using the Kaplan–Meier method, and differences were assessed by the log-rank test. A *P*-value of less than 0.05 was taken to indicate a statistically significant difference.

## RESULTS

Both relative TS and DPD mRNA expressions were determined by the fluorescence-based real-time detection method in specimens from all 53 patients with obtained the primary gastric cancer. Tumours were categorised as either responding or not responding to each regimen.

**Figure 1:**
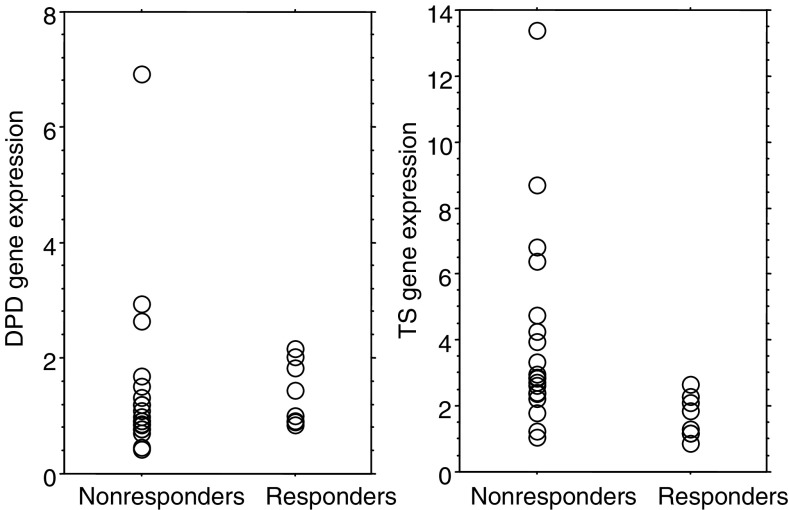
DPD and TS mRNA in 27 primary gastric cancer treated with S-1 alone in relation to nonresponse or response. There was no statistical difference in DPD gene expression among responding and nonresponding tumours. Median values of TS mRNA were 1.58 and 2.88 for responding tumours and nonresponding tumours, respectively (Mann–Whitney *U* test; *P*<0.005).

In the S-1-alone regimen, the median values of DPD mRNA expressions were 1.22 (range: 0.84–2.16) and 0.99 (range: 0.42–6.93) for responding tumours and nonresponding tumours, respectively, without a statistically significant difference (*P*=0.41; Mann–Whitney *U* test; Figure 1

). In contrast, the median value of TS mRNA expression in responding tumours was 1.58 (range: 0.85–2.66). It was significantly lower than the 2.88 value (range: 1.06–13.37) in nonresponding cases (*P*<0.005; Mann–Whitney *U* test; Figure 1).

The median value of TS in patients treated with the S-1-alone regimen was 2.61 (range: 0.85–13.37), which was selected for a cutoff value to separate high and low gene expression of TS. In patients treated with S-1 alone, response rates were 50% (seven out of 14) and 8% (one out of 13) in low (TS ⩽2.61) and high TS (TS >2.61) tumours, respectively (*P*<0.05; two-sided Fisher's exact test) (Table 2

**Table 2:** Summary of response for tumours according to TS gene expression in patients treated with S-1 alone or S-1 combined with CPT-11

	**TS⩽2.61**	**TS>2.61**	
*S-1 alone*			
No. of responding patients	7	1	
No. of nonresponding patients	7	12	
*P*-value			<0.05
			
	**TS ⩽2.54**	**TS >2.54**	
*S-1 plus CPT-11*			
No. of responding patients	5	8	
No. of nonresponding patients	8	5	
*P*-value			0.43

). Patients with low TS gene expression survived longer than those with high TS gene expression, with statistical significance (median: 9.3 months ranging from 3.2 to 23.0 months for patients with low TS gene expression tumours *vs* 4.0 months ranging from 2.1 to 8.6 months for patients with high TS gene expression tumour, *P*<0.0001; log-rank test; Figure 2

**Figure 2:**
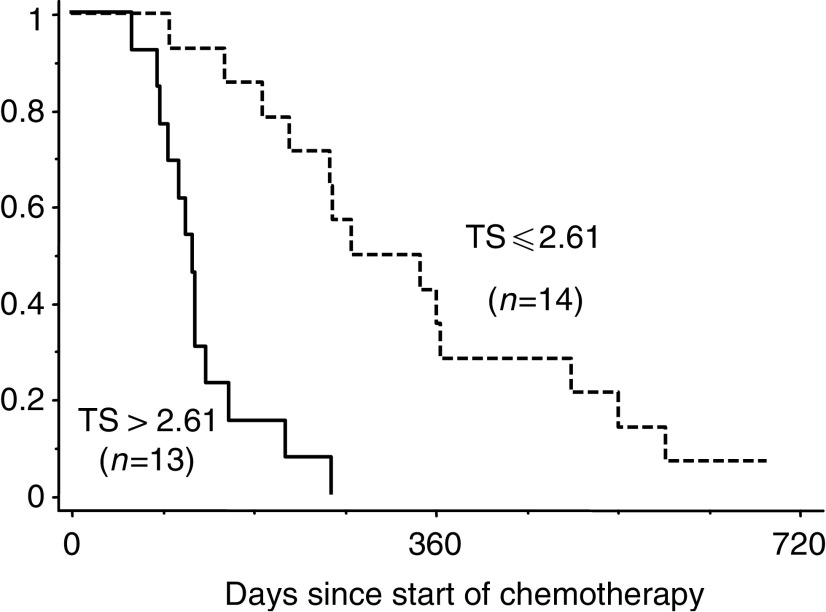
Cumulative survival curves (Kaplan–Meier) when treated with S-1 alone.

).

**Figure 3:**
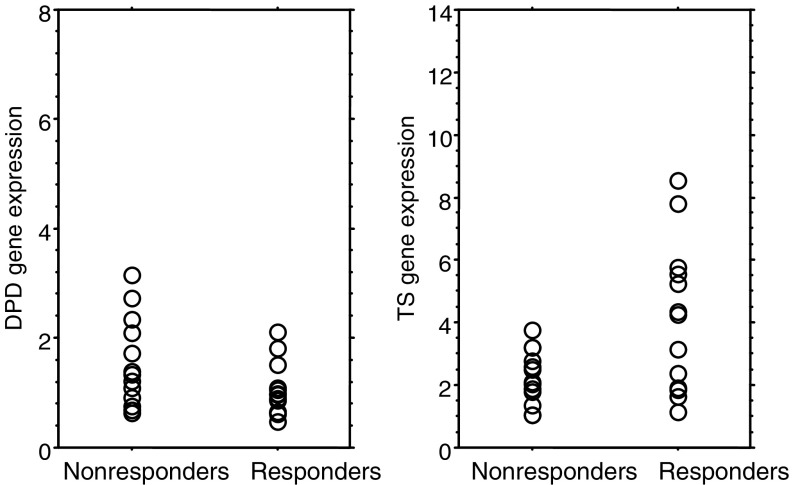
DPD and TS mRNA in 26 primary gastric cancer treated with S-1+CPT-11 in relation to nonresponse or response. There was no statistical difference in DPD gene expression among responding and nonresponding tumours. Median values of TS mRNA expression in responding and nonresponding tumours were 4.26 and 2.11, respectively, with a trend in favour of a higher TS mRNA expression for responding tumours (*P*=0.055; Mann–Whitney *U* test).

In the S-1 combined with CPT-11 group, DPD mRNA expressions showed no statistical difference between responding (median 0.97, ranging from 0.48 to 2.12) and nonresponding tumours (median 1.34, ranging from 0.64 to 3.16) (*P*=0.10; Mann– Whitney *U* test; Figure 3

). Median values of TS mRNA expression in responding and nonresponding tumours were 4.26 (range: 1.13–8.54) and 2.11 (range: 1.04–3.77), respectively (*P*=0.055; Mann–Whitney *U* test; Figure 3).

**Figure 4:**
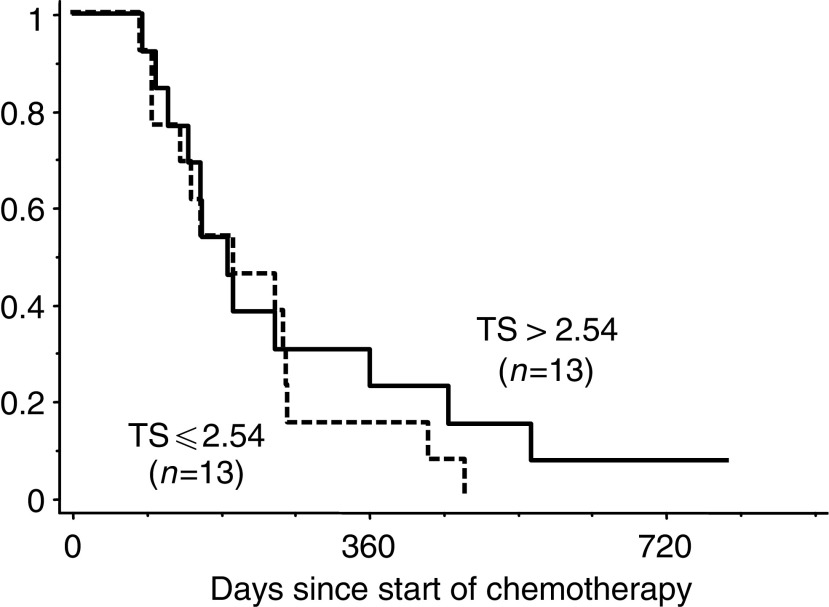
Cumulative survival curves (Kaplan–Meier) when treated with S-1 combined with CPT-11 in patients.

The median value of TS in patients treated with the S-1 combined with CPT-11 regimen was 2.54 (range: 1.04–8.54), which was selected for a cutoff value to separate high and low gene expression of TS. The response rates were 38% (five out of 13) and 62% (eight out of 13) in tumours with low (TS ⩽2.54) and high TS (TS >2.54) gene expression, respectively, with no statistical significance (*P*=0.43; two-sided Fisher's exact test) (Table 2). The median survival times were 6.6 months ranging from 2.8 to 16.0 months and 6.3 months ranging from 2.8 to 26.6 months for patients with low TS gene expression tumour and those with high TS ones, respectively, without statistical significance (*P*=0.507; log-rank test; Figure 4

).

## DISCUSSION

In this study, we demonstrated that high TS gene expression in primary gastric cancer predicted poor response in metastatic tumour, with shorter survival, when treated with S-1 alone. When CPT-11 was combined with S-1, intratumour TS gene expression had no predictive values in terms of tumour shrinkage and survival. Our data also demonstrated that the antitumour effect of S-1 for gastric cancer was not influenced by intratumour DPD gene expression, with or without combination with CPT-11.

TS expression in a primary gastric cancer had correlated with the response of the primary tumour or metastatic tumour and survival when treated by 5-FU containing regimen (Lenz *et al*, 1995; Boku *et al*, 1998; Yeh *et al*, 1998). As in previous reports, tumours with low TS gene expression also had better response, and longer survival than those with high TS gene expression when treated with S-1 alone. (Figures 1 and 2).

However, experiments using human gastric cancer xenografts indicated that there was no correlations between the antitumour effect of S-1 and TS enzymatic activity and that an enhanced blockade of RNA function attributed to the cytotoxicity of S-1 in the addition of an increased inhibition of DNA (Fujiwara *et al*, 2003). S-1 showed antitumour effects in terms of response and survival, regardless of the expression status of TS, when TS expression was evaluated by immunohistochemical method using antirecombinant human TS polyclonal antibody (Miyamoto *et al*, 2000). These findings did not agree with our results. The reason for this disagreement among results remains unclear. The discrepancy could therefore be due to the xenograft model, in which S-1 was administered orally at doses of 10 mg kg^−1^. Another possible explanation might be the difference in methodology for measuring TS expression, because the fluorescence-based real-time detection method is a more quantitative and objective evaluation method than the immunohistochemical method (Kornmann *et al*, 2003).

When treated with S-1 combined with CPT-11, TS gene expression does not predict the antitumour effect (Table 2). In the CPT-11 combined with S-1 group, responding tumours had a nonstatistically significant tendency towards higher TS gene expression, compared with nonresponding ones (Figure 3). These data suggested that tumours with a high expression of TS might respond to additional CPT-11, whereas those tumours were refractory to S-1 alone.

While the gene expression of Topo-I was reported to predict tumour response to camptothecin derivative in cell cultures (McLeod and Keith, 1996), clinically the predictive value of Topo-I gene expression is still controversial (Vanhoefer *et al*, 2001). A positive relation between TS and Topo-I mRNA expression was observed in colorectal cancer tissue (Ichikawa *et al*, 1999). The response rates to CPT-11 alone had been reported to be 43 and 15% in tumours with high and low TS expression, respectively, among a group of colorectal cancer patients that did not respond to 5-FU (Danenberg, 2003). The relationship between TS and Topo-I mRNA expression in gastric cancer is unclear and further studies are necessary to define the molecular mechanisms underlying the regulation of these genes in gastric cancer.

Intratumoural DPD expression inversely correlates with the sensitivity to 5-FU (Ishikawa *et al*, 1999; Nozawa *et al*, 2002) in gastric cancer. However, our data indicated that the antitumour effect of S-1 for gastric cancer was not influenced by intratumour DPD gene expression, regardless of the combination of CPT-11. This difference was explained by the inhibition of intratumoural DPD by CDHP, which is contained in S-1 (Takechi *et al*, 2002). There were no correlations between the antitumour activity of S-1 and DPD activity in human gastric xenografts (Fujiwara *et al*, 2003). Miyamoto *et al*. also reported that patients with positive DPD showed a slightly higher response rate and longer survival than those with negative DPD, but without statistical significance, when DPD expression was evaluated by immunohistochemical methods using antirecombinant human polyclonal antibody (Miyamoto *et al*, 2000). S-1 is thought to have antitumour activity even in highly DPD expressed tumour, which is essentially resistant to fluoropyrimidine without DPD inhibitor (Salonga *et al*, 2000; Ichikawa *et al*, 2003).

TS and DPD gene expressions in primary gastric cancer differed according to degree of differentiation (Ichikawa *et al*, in press). TS gene expression was statistically higher in intestinal type than diffuse type in 78 gastric cancer tissues, whereas DPD gene expression of diffuse type was statistically higher than that of intestinal type. The same results were obtained in this study cohort including 53 gastric cancer tissues (data not shown). In a phase II study of S-1 for metastatic gastric cancer, the response rate of the diffuse type was higher than that for the intestinal type (Koizumi *et al*, 2000). A higher response rate of S-1 for diffuse type might be explained by both low TS expression and the inhibition of high DPD by CDHP (Takechi *et al*, 2002; Fujiwara *et al*, 2003). Because this study involved only a small number of patients, we could not evaluate the predictive values of histological type when combined with TS and DPD gene expression.

In this study, the S-1 combined with CPT-11 achieved the high response rate of 50.0%, in comparison to the response rate of 29.6% in the S-1-alone regimen. However, median survival times were 6.3 months in both regimens. In the randomised phase III trial in patients with metastatic gastric cancer, the median survival times ranged from 7.1 to 7.3 months, although the response rates were statistically significant; 11% for the 5-FU-alone regimen and 34% for 5-FU combined with CDDP (Ohtsu *et al*, 2003). Thus, it is not surprising that a higher response rate was not related with longer survival time in the chemotherapy for gastric cancer (Ajani, 2000). Another possible explanation is the difference of second-line treatment regimens (Table 1). Interestingly, in patients with low TS expressed tumors, the response rate and survival for S-1 combined with CPT-11 were both worse than for the S-1-alone regimen (response rate and median survival time; 38% and 6.6 months for S-1 combined with CPT-11 and 50% and 9.3 months for S-1 alone). This might be explained by the decreased dose intensity of S-1 in the S-1 combined with CPT-11 regimen, compared with the S-1-alone regimens. In the S-1 combined with CPT-11 regimens, S-1 is given for 2 weeks with 2 weeks' rest, although in the S-1-alone regimen there is consecutive administration for 4 weeks followed by 2 weeks of no drug administration.

In conclusion, treatment effect of S-1 for gastric cancer determined by the status of TS gene expression, regardless of that of DPD gene expression. When CPT-11 combined with S-1, intratumour TS gene expression did not predict antitumour effect. However, these conclusions have been drawn from a limited retrospective study of a relatively small number of patients. Prospectively, randomised, translational treatment trials are needed to corroborate our results.
